# The Janus-faced functions of Apolipoproteins L in membrane dynamics

**DOI:** 10.1007/s00018-024-05180-9

**Published:** 2024-03-13

**Authors:** Etienne Pays

**Affiliations:** Laboratory of Molecular Parasitology, IBMM, Université Libre de Bruxelles, 6041 Gosselies, Belgium

**Keywords:** Membrane fission, Membrane fusion, Secretion, Mitophagy, Autophagy, Apoptosis, Kidney disease, Sleeping sickness, Adipocyte adipogenesis, APOL1 risk variants

## Abstract

The functions of human Apolipoproteins L (APOLs) are poorly understood, but involve diverse activities like lysis of bloodstream trypanosomes and intracellular bacteria, modulation of viral infection and induction of apoptosis, autophagy, and chronic kidney disease. Based on recent work, I propose that the basic function of APOLs is the control of membrane dynamics, at least in the Golgi and mitochondrion. Together with neuronal calcium sensor-1 (NCS1) and calneuron-1 (CALN1), APOL3 controls the activity of phosphatidylinositol-4-kinase-IIIB (PI4KB), involved in both Golgi and mitochondrion membrane fission. Whereas secreted APOL1 induces African trypanosome lysis through membrane permeabilization of the parasite mitochondrion, intracellular APOL1 conditions non-muscular myosin-2A (NM2A)-mediated transfer of PI4KB and APOL3 from the Golgi to the mitochondrion under conditions interfering with PI4KB-APOL3 interaction, such as APOL1 C-terminal variant expression or virus-induced inflammatory signalling. APOL3 controls mitophagy through complementary interactions with the membrane fission factor PI4KB and the membrane fusion factor vesicle-associated membrane protein-8 (VAMP8). In mice, the basic APOL1 and APOL3 activities could be exerted by mAPOL9 and mAPOL8, respectively. Perspectives regarding the mechanism and treatment of APOL1-related kidney disease are discussed, as well as speculations on additional APOLs functions, such as APOL6 involvement in adipocyte membrane dynamics through interaction with myosin-10 (MYH10).

## Introduction

The APOL multigene family is highly dynamic, and encodes 6 human and 12 mouse protein members (phylogenetic trees in [1, 2]). However, detailed information on APOL function is only available for some members (human APOL1 and APOL3, mouse APOL6, APOL8 and APOL9) [3]. In this family, the primate-specific APOL1 is the only member containing an N-terminal signal peptide for secretion [3]. Secreted APOL1 kills the African bloodstream parasite *Trypanosoma brucei brucei* [4, 5]. Uptake of APOL1 triggers trypanosome lysis through lysosomal and mitochondrial membrane permeabilization due to APOL1 pore-forming activity in parasite endosomal membranes, followed by kinesin-mediated transport of the APOL1-carrying endosomes to the mitochondrion [6]. Two *T. brucei* subspecies termed *T. b. rhodesiense* and *T. b. gambiense* acquired resistance to APOL1, enabling these parasites to infect humans and cause sleeping sickness, in Eastern and Western Africa respectively [5]. In Western Africa, two C-terminal APOL1 variants called G1 and G2 (respectively S342G/I384M mutations and 388NY389 deletion) are widespread. Both variants exhibit in vitro killing activity on *T. b. rhodesiense* [7], and in the field, G2 is linked to full resistance to *T. b. rhodesiense* whereas G1 provides partial resistance to both *T. b. rhodesiense* and *T. b. gambiense* [8]. Thus, APOL1 initially allowed humans to survive infection by *T. b. brucei*, but afterwards an evolutionary arms race generated APOL1 variants to counteract acquired parasite resistance to APOL1 [5].

Besides resistance to *T. b. rhodesiense* infection, humans expressing the G1 or G2 variants exhibit increased susceptibility to chronic kidney disease, and this was reproduced in G1- or G2-expressing transgenic mice [7, 9, 10]. This disease results from kidney dysfunctions linked to changes of podocyte architecture and motility, accompanied by reduction of autophagic flux [10, 11].

In contrast to APOL1, APOL3 is not secreted [12], and kills intracellular bacteria through detergent-like activity on bacterial membranes [13]. Despite their differential activities, APOL1 and APOL3 share strongly increased expression under inflammatory conditions associated with resistance to infection [3].

Until recently, these observations represented the only salient information available regarding APOL function, apart from pleiotropic cellular toxicity of the APOL1 variants, reported in experimental studies mostly involving ectopic gene expression [14, 15]. Altogether, these observations linked APOLs to cellular killing or toxic activities. However, recent work [16] has uncovered the crucial involvement of APOL1 and APOL3 in intracellular membrane fission and fusion, suggesting that their basic function is not to induce pathogen lysis, but to control membrane dynamics.

### APOL1 and APOL3 structure

**Fig. 1:**
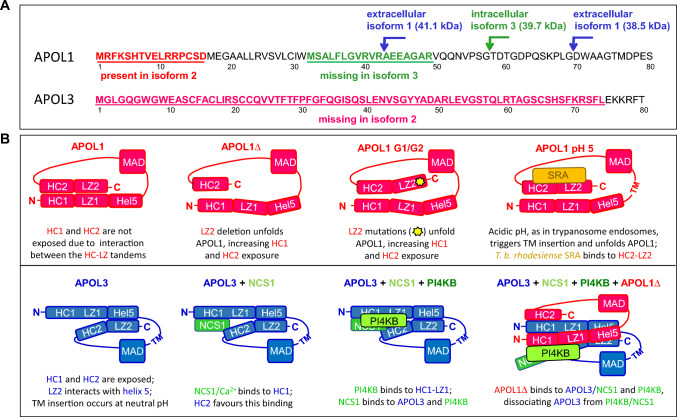
Processing, folding and interactions of APOL1 and APOL3. **A** N-terminal region of the sequences encoded by the APOL1 and APOL3 genes, with alternative mRNA splicing-dependent generation of isoforms. In APOL1, peptide addition (red, isoform 2) or deletion (green, isoform 3) allows the distinction with isoform 1. The N-terminal cleavage sites are indicated in APOL1 isoform 1 (blue arrows) and isoform 3 (green arrow). Regarding APOL3, isoform 2 (36.5 kDa) is the main version found in podocytes [11]. **B** Models of *cis*- and *trans*-interactions of APOL1 and APOL3 (HC = hydrophobic cluster; Hel = helix; LZ = leucine zipper; MAD = membrane-addressing domain; TM = transmembrane span; yellow asterisk = G1 and G2 mutations). For sake of clarity each HC-LZ tandem is presented as a linear structure, but these tandems, as well as MAD, are probably folded as double stranded hairpins

APOL1 and APOL3 gene transcripts are alternatively spliced in their 5’-terminal region, generating mRNAs variants that encode different isoforms (Fig. 1A). Whereas APOL1 isoform 1 is secreted (with two N-terminal cleavage sites), APOL1 isoform 3 and APOL3 isoform 2 represent the main intracellular versions [11]. The hypothetical structures of APOL1 and APOL3 are schematized in Fig. 1B. The N-terminal domain of secreted APOL1 contains 5 helices including a non-classical four-helix bundle (helices 2–5) that is also present in APOL3 [17]. Helices 2 and 4 correspond to stretches respectively defined as hydrophobic cluster 1 (HC1) and leucine zipper 1 (LZ1) in Uzureau et al. [11]. Downstream from the N-terminal domain, both proteins contain a hydrophobic hairpin helix potentially able to cross a membrane (TM, for transmembrane). In case of APOL1, acidic residues in these helices only allow TM insertion under acidic conditions, these conditions being absolutely required for trypanosome killing [6, 12]. The TM hairpin is immediately adjacent to another helix hairpin termed MAD for Membrane-Addressing Domain, because in experimental studies in *Escherichia coli* this domain was necessary for membrane association of APOL1 [18]. This domain was also partially protected against protease degradation upon in vitro APOL1 incubation with isolated mitochondrial membranes [6], confirming its tight association with membranes. The MAD domain could be involved in APOL1 and APOL3 binding to cardiolipin [11], the characteristic phospholipid of both bacterial and mitochondrial membranes. Accordingly, APOL1 associates with the mitochondrion when expressed in yeast [19]. After a long hinge sequence (APOL1 A291-A339; APOL3 A232-T272), a second HC-LZ tandem (HC2-LZ2) characterizes the C-terminal region of both APOLs (APOL1 S342-L392; APOL3 G275-L325). In case of APOL1 but not APOL3, the HC2-LZ2 tandem strongly interacts with HC1-LZ1, imposing a specific protein folding that underlies differential *trans*-interactions of APOL1 with respect to APOL3 [11, 16].

As schematized in Fig. 1B, *cis*-interaction between the two APOL1 HC-LZ regions prevents HCs exposure. In case of LZ2 deletion or mutations, as occurs for experimental C-terminally truncated APOL1Δ (stop at V353) or natural G1/G2 C-terminal variants respectively, *cis*-interaction is disrupted and APOL1 hydrophobicity is increased due to HC exposure [11]. This is linked to enhanced APOL1 interaction with APOL3, resulting in APOL3 inactivation phenotypically mimicking APOL3 deletion [11]. Under acidic conditions as in trypanosome endosomes, APOL1 can insert into membranes and *cis*-interaction between the two HC-LZ regions is lost [11, 16]. In endosomes of *T. b. rhodesiense*, the HC2-LZ2 tandem interacts with the Serum Resistance-Associated protein SRA, which neutralizes APOL1 and prevents trypanosome lysis [4, 5]. In APOL3, no *cis*-interaction between HC-LZ regions occurs, but LZ2 may interact with helix 5 [16]. In striking contrast with APOL1, APOL3 tightly binds to the Ca^2+^ sensor protein NCS1 and the PI4KB kinase, and this involves HC1 [16]. However, C-terminal APOL1 variants can bind to PI4KB, owing to increased HC1 exposure (strongly for APOL1Δ, weakly for G1 or G2) [16].

### APOL1 and APOL3 pore-forming activity

In helix 5, APOL1 and APOL3 contain a peptide resembling the BCL-homology-3 (BH3) motif typical of the apoptotic BCL2 (B-cell lymphoma 2) family, and like BCL2 family members, APOL1 and APOL3 contain a TM hairpin responsible for in vitro membrane pore-forming and cellular killing activities [6, 11, 12, 18]. In trypanosomes, APOL1 and APOL3 induce apoptotic-like cell lysis due to permeabilization of the parasite mitochondrial membrane followed by translocation of a mitochondrial endonuclease into the nucleus, causing DNA fragmentation [6, 12]. Interestingly, APOL1- and APOL3-mediated trypanosome killing is accompanied by increased fusion between mitochondrial membranes [6, 12]. In either mouse dendritic cells or human podocytes, APOL1 and APOL3 are involved in apoptosis induced by interferon-I (IFN-I)-mediated inflammation [11, 20]. Given the key involvement of the mitochondrial membrane in apoptosis, the activity of these APOLs is thus linked to modifications of this membrane.

**Fig. 2:**
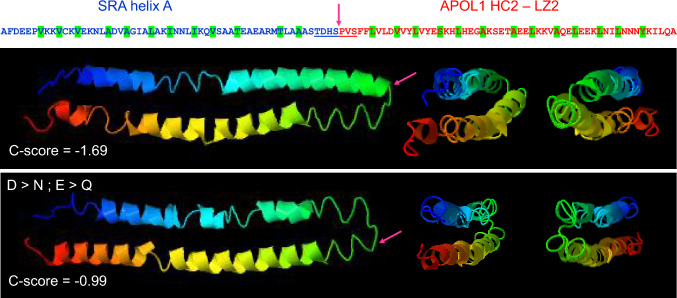
Models of SRA interaction with HC2-LZ2 under neutral or acidic conditions. I-TASSER modelling (https://zhanggroup.org/I-TASSER/) of an artificial polypeptide construct linking the SRA helix A (A31-S85) with the APOL1 HC2-LZ2 region (P340-A394), for evaluation of their interaction potential. The link (arrowed) is set after the helix A downstream loop and before the HC2 upstream loop (loops are underlined). Hydrophobic residues of heptad repeats are highlighted in green. In the bottom panel, all D and E residues were replaced by N and Q, respectively, to mimic protonation of negatively charged amino acids occurring under acidic conditions in trypanosome endosomes. The C-score allows the evaluation of the model quality (between − 5 and + 2; higher value = higher confidence). The best prediction is shown for each condition

Cell culture studies, mostly involving ectopic APOL1 expression, suggested that intracellular APOL1 variants trigger pleiotropic toxicity [14, 15]. However, experimental artefacts linked to APOL1 overexpression and/or mislocalization cast important doubts regarding the physiological relevance of these observations [3, 21–23]. Intracellular APOL1 activities are distinct from extracellular ones, because these activities are performed by different APOL1 isoforms, at different pHs and with a different topology with respect to the target membrane [22]. In trypanosomes, APOL1 killing activity was clearly due to the central hairpin helix, whose transmembrane insertion requires a low pH [6, 12]. In trypanosome endosomes, APOL1 triggers weak transmembrane anionic fluxes responsible for lysosome swelling [18], but under neutral conditions the APOL1 pore exhibits strong cationic conductance ascribed to negatively charged residues present in HC2 [24, 25]. Therefore, it was proposed that the HC2-LZ2 region is involved in cation conductance at the plasma membrane after prior HC2 insertion into endosomal membranes [26, 27]. Transmembrane HC2 insertion can only occur under acidic conditions because it requires protonation of the acidic HC2 residues, coupled to acidic pH-mediated disruption of *cis*-interaction between the two HC-LZ tandems [16, 22]. However, such insertion is hardly compatible with the known presentation of HC2-LZ2 in the lumen of trypanosome endosomes, where this helix tandem can interact with the N-terminal helix A of *T. b. rhodesiense* SRA, as determined by both experimental evidence and in silico modelling [4, 22] (Fig. 2). Moreover, the strong membrane disrupting activity of HC2 [4] is unlikely to allow stable channel formation in the plasma membrane. Finally, transmembrane HC2 insertion cannot occur at neutral pH in APOL3, and this impossibility does not prevent APOL3 from exhibiting fully pH-independent cationic pore-forming activity in vitro, as well as fully pH-independent trypanosome-killing ability [12]. Therefore, cationic selectivity of the pore could result not from membrane insertion of HC2, but from positioning of conserved HC2 acidic residues close to the cytoplasmic pore entry site, which would be affected by either HC2-LZ2 interaction with SRA or LZ2 deletion as occurs in APOL1Δ [22]. Irrespective of the eventual transmembrane insertion of HC2, APOL1 ionic pore-forming activity is unlikely to account for the specific effects of APOL1 variants in podocytes because intracellular APOL1 was not detected within podocyte acidic compartments. In vitro*,* a twofold difference of K^+^ pore-forming activity was identified between wild-type (WT) APOL1 and C-terminal variants [28], but whether such difference is sufficient for inducing in vivo toxicity remains uncertain.

Contrasting with experiments involving ectopic APOL1 expression, studies involving CRISPR/Cas9-mediated gene editing in podocytes did not reveal generalized toxicity of the C-terminally-truncated APOL1 variant APOL1Δ. These studies rather revealed changes in organization of actomyosin linked to reduction of Golgi PI4KB activity, with effects on cell shape and motility as well as on Golgi and mitochondrion structure. This phenotype strikingly resembled that of natural G1- or G2-expressing podocytes, albeit with increased severity as expected from the difference between full LZ2 deletion and only mutations [11]. APOL1Δ strongly reduced apoptosis and inhibited in vitro pore-forming activity of APOL3-NCS1 complex following direct interaction with this complex [11]. Importantly, APOL1Δ did not exert ionic pore-forming activity in vitro or cellular toxicity in trypanosomes [11, 24, 29], clearly indicating that the severe G1/G2-like phenotype of APOL1Δ-expressing podocytes cannot be ascribed to APOL1-driven ionic pore formation. Consistently, trypanosome-killing activity of APOL1 or APOL3 appeared to result not from cationic fluxes, but from mitochondrial megapore formation, as occurs for apoptosis in higher eukaryotic cells [6, 12, 22]. Along this line, like APOL1 or APOL3 the apoptotic BCL2 family members also exhibit ionic pore-forming activity in vitro and cellular toxicity upon ectopic expression, but in vivo these proteins form lipidic rather than proteinaceous oligomeric pores, and so far, no evidence involves ionic pore-forming activity in apoptosis [30, 31].

A recent model of APOL1-mediated podocytopathy proposes that upon IFN-I signalling, cationic pores formed by APOL1 variants at the plasma membrane induce intracellular Ca^2+^ fluxes through activation of G protein-coupled receptors (GPCR) and inositol 1,4,5-trisphosphate receptors (IP3R) [32]. However, the existence of plasma membrane APOL1 pores remains to be demonstrated, and the surface cationic fluxes were not proven to occur through such pores. Since both NCS1 and the PI4KB product phosphatidylinositol-4-phosphate (PI4P) are involved in cation channel activity at the plasma membrane, including K^+^ efflux by Kv4 channels [33–35], delocalizing PI4KB and NCS1 activities from the Golgi as occurs upon APOL3-PI4KB complex disruption [11, 16] could affect surface cation fluxes independently of APOL1 pores. In addition, and importantly, the intracellular Ca^2+^ fluxes could result not from APOL1 pore activity, but from increased NCS1 binding to GPRCs and GPCR kinases [36–39] as well as NCS1 binding to IP3Rs, including at endoplasmic reticulum-mitochondrion contact sites (MERCSs) [33, 39, 40]. Indeed, NCS1 controls the activity of both receptor types, and NCS1 delocalization from the Golgi may alter the activity of these receptors at both plasma and endoplasmic reticulum membranes. The demonstration that the APOL1 inhibitor VX-147 prevents both APOL1 variant-induced toxicity and K^+^ fluxes [32] does not prove that APOL1 variant-driven cationic pore formation is responsible for kidney disease, because VX-147 has not been demonstrated to specifically block the APOL1 pore, but could alternatively interfere with APOL1 folding, interactions and/or stability, inhibiting other processes affected by APOL1 variants.

In conclusion, APOL1 cationic pore-forming activity can be observed in vitro, but is unlikely to occur at the plasma membrane in vivo, and all phenotypic consequences of APOL1 C-terminal variants expression can be explained by the effects of APOL3 inactivation on PI4KB and NCS1 trafficking, rather than APOL1 pore-forming activity. Thus, kidney disease induced by APOL1 risk variants may result not from intrinsic APOL1 toxicity, but rather from improper cell function and consecutive inflammatory reaction.

### APOL3 controls membrane fission through interaction with PI4KB

**Fig. 3:**
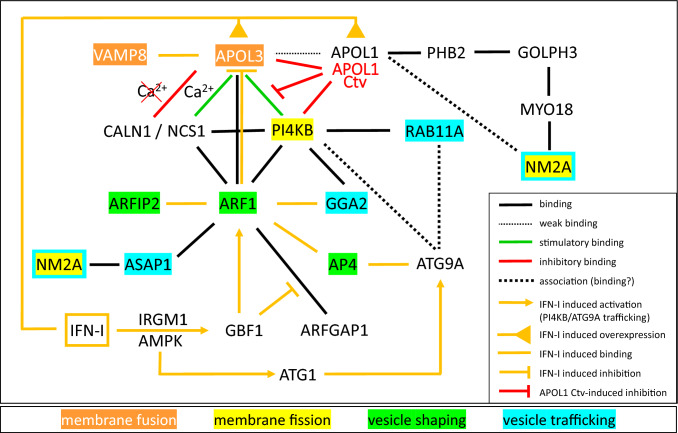
Model of the PI4KB interactome. The scheme shows interactions between various proteins known to bind to PI4KB and/or to affect PI4KB localization and activity at the Golgi, including interactions induced or inhibited by IFN-I signalling, like occurs following viral infection. AP4: Adaptor-protein-complex-4; APOL1 Ctv: APOL1 C-terminal variants; ARFIP2: Arfaptin-2; ASAP1: Arf-GAP-with-SH3-domain-ANK-repeat-and-PH domain-containing-protein-1; GGA2: Golgi associated, gamma adaptin ear containing, ARF binding protein 2; RAB11A: Ras-related protein 11A. Other abbreviations: see the text. All proteins mentioned here associate at least transiently with the Golgi membrane, with dynamic trafficking that particularly involves ATG9A vesicles

**Fig. 4:**
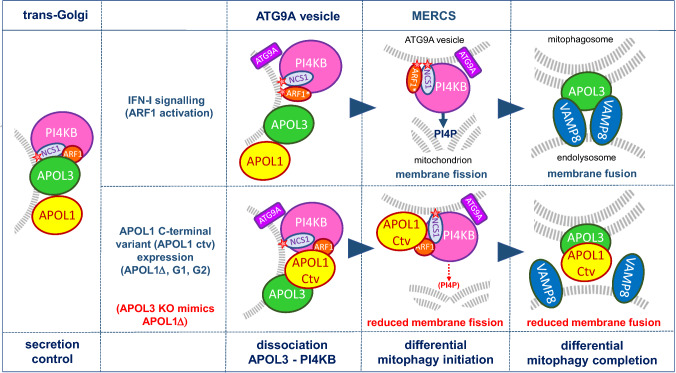
Hypothetical model of the APOL3 and APOL1 activities. APOL1 and APOL3 are present at the trans-Golgi membrane in association with NCS1 and PI4KB. PI4KB activity promotes membrane fission for secretion and exocytosis. IFN-I signalling triggers increased expression of APOL1 and APOL3, and affects APOL3 interaction with PI4KB and NCS1 following the binding of activated ARF1 to PI4KB/NCS1. This allows PI4KB and NCS1 delocalization. Similarly, APOL1 C-terminal variants (APOL1 Ctv) can induce APOL3 dissociation from PI4KB, partially or totally mimicking APOL3 deletion (G1/G2 or APOL1Δ, respectively). PI4KB is trafficked in NM2A-driven ATG9A vesicles derived from the Golgi, which also carry APOL3 and APOL1. ATG9A vesicles are targeted to MERCSs, and this may involve APOL1 binding to the mitophagy receptor PHB2. The PI4KB-ARF1 complex of ATG9A vesicles triggers mitochondrial fission and mitophagy through the formation of PI4P-enriched rafts that allow the recruitment of fission factors such as NM2A, GOLPH3 and FIS1. Mitochondrion fission is followed by mitophagosome formation. In either APOL3-KO cells or APOL1 Ctv-expressing cells (G1, G2 or APOL1Δ), PI4KB inhibition resulting from lack of ARF1 activation reduces membrane fission and mitophagosome formation. Given its strong binding to cardiolipin, APOL3 could be recruited to the mitophagosome membrane. Fusion of mitophagosomes with endolysosomes is ensured by interaction between APOL3 and VAMP8, which involves APOL3 hairpin helices on both sides of the transmembrane hairpin (helices 4–5 and MAD1-2). The interaction of APOL1 Ctvs with APOL3 prevents this fusion, inducing cellular damage due to mitochondrial dysfunction. The red stars symbolize the N-linked myristate, not exposed in ARF1-GDP. ARF1* = activated ARF1 (ARF1-GTP)

Strikingly contrasting with APOL1, APOL3 was found to exhibit high-affinity interactions with PI4KB, NCS1, CALN1 and ADP-ribosylation-factor-1 (ARF1) [16]. All these proteins participate in PI4P synthesis at the *trans*-Golgi, NCS1 and CALN1 respectively increasing and decreasing PI4KB activity depending on the presence of Ca^2+^ [41]. APOL3, but not NCS1, stimulated in vitro PI4KB activity provided Ca^2+^ was present, and conversely CALN-1 inhibited PI4KB in the absence of Ca^2+^ [16]. In vivo, NCS1 or CALN1 interaction with APOL3 may allow their association with the Golgi membrane, NCS1 providing the local Ca^2+^ needed for PI4KB activation by APOL3. Together with factors controlling vesicle shaping and trafficking, the different proteins interacting with APOL3 participate in secretion and exocytosis (Fig. 3). These processes are influenced by interferon-I (IFN-I) signalling, as occurs upon viral infection. IFN-I-induced Immunity-related-GTPase-family-M-1 (IRGM1) triggers both Golgi fragmentation and autophagy through activation of the AMP-activated-protein-kinase (AMPK), which phosphorylates both the Golgi-specific-brefeldin-A-resistance-guanine-nucleotide-exchange-factor-1 (GBF1) and the Autophagy-1 kinase (ATG1) [42]. While GBF1 induces ARF1 dissociation from ADP-ribosylation-factor-GTPase-activating protein-1 (ARFGAP1) and activates ARF1 (GTP binding), ATG1 initiates autophagy at MERCS pre-autophagosomal structures, inducing the coalescence of Golgi-derived vesicles carrying the lipid scramblase autophagy-related-protein-9A (ATG9A). At the Golgi, ARF1 activation promotes its membrane recruitment and increased binding to PI4KB, possibly disrupting APOL3 interaction with the PI4KB/NCS1 complex [16]. IFN-I signalling results in PI4KB/NCS1/ARF1 delocalization from the Golgi and trafficking to the mitochondrion in vesicles carrying ATG9A together with APOL1 and APOL3, in a process triggering mitophagy (Fig. 4) [3, 16].

In human podocytes, either natural or CRISPR/Cas9-mediated APOL3 KO mimicked the G1/G2 phenotype, but with higher severity [11, 43]. Absence of APOL3 led to strong reduction of PI4KB activity at the Golgi, Golgi shrinkage and concomitant reduction of mitochondrial membrane fission [11, 16]. I propose that this phenotype results from PI4KB transfer to the mitochondrion following PI4KB release from the Golgi membrane like occurs upon ARF1 activation, but with reduced PI4KB fission-inducing activity due to lack of ARF1 activation (Fig. 4). Indeed, PI4KB is required with ARF1 for mitochondrial membrane fission [44–49]. Accordingly, the involvement of IP3R-induced Ca^2+^ efflux in triggering mitochondrial fission at MERCSs [40, 50] probably reflects NCS1/Ca^2+^-mediated activation of PI4KB. The PI4KB product PI4P binds to the NM2A myosin and membrane-bending factors linked to actin polymerization at the fission site [44, 51–53]. PI4P can oligomerize into lipid raft-like domains that recruit Golgi-phosphoprotein-3 (GOLPH3), a *trans*-Golgi and mitochondrial protein binding to the F-actin and NM2A binder myosin-18A (MYO18) to generate a pulling force for membrane fission [54–56]. Other potential PI4P-binding fission components involved in auto/mitophagy include fission-1 (FIS1) and syntaxin-17 (STX17) [57–60].

I conclude that PI4KB activity conditions membrane fission at the Golgi and mitochondrion, in a process controlled by APOL3 and activated ARF1, at the Golgi and mitochondrion respectively.

### C-terminal APOL1 variants disrupt APOL3-PI4KB interaction at the Golgi

The APOL1 C-terminal variants (natural G1 and G2, or experimental APOL1Δ) were found to exhibit increased binding to both APOL3 and PI4KB, affecting the interaction of APOL3 with PI4KB and therefore, reducing Golgi PI4P synthesis by PI4KB [11, 16]. Binding to PI4KB may trigger its release from Golgi membrane anchoring because unlike APOL3, the APOL1 variants cannot insert into membranes at a neutral pH. Accordingly, in APOL1Δ-expressing podocytes PI4KB was dissociated from APOL3, mimicking the effect of APOL3 KO [11]. Thus, APOL1 unfolding due to alteration or deletion of the C-terminal LZ2, as occurs in G1, G2 or APOL1Δ podocytes, results in relative disruption of APOL3-PI4KB interaction, inducing a similar dysfunctional phenotype linked to PI4KB delocalization from the Golgi, although with differing intensities.

Mitochondria appeared to fragment upon either APOL3 KO or APOL1Δ, G1 or G2 expression, suggesting that APOL3 loss or inactivation triggers mitochondrial fission [11, 61]. While consistent with the view that PI4KB delocalizes to the mitochondrion upon disruption of the APOL3-PI4KB complex, this observation seems to contradict the proposal presented in the previous paragraph, that under these conditions, the PI4KB fission-inducing activity is reduced due to lack of ARF1 activation. The data presented in Lecordier et al. [16] provide an explanation for this apparent discrepancy: mitochondrial fragmentation in APOL3 KO or APOL1Δ cells does not result from increased fission, but from accumulation of non-digested mitophagosomes (see below).

### APOL3 induces membrane fusion through interaction with VAMP8

In either APOL3 KO or APOL1Δ-expressing podocytes, the mitophagy flux was found to be reduced not only because of decreased mitochondrion fission, but also because of decreased mitophagosome fusion with endolysosomes, leading to the accumulation of dark, convoluted and multilamellar mitophagosomes, particularly in APOL1Δ cells [16]. Decreased vesicle fusion following APOL3 inactivation suggested that APOL3 is involved in membrane fusion. Accordingly, increased mitochondrial membrane fusion was observed following the uptake of APOL3 or APOL1 in trypanosomes [6, 12] and APOL3 exhibited fusion activity on membranes of intracellular bacteria [13]. Thus, APOL3 and APOL1 appear to induce membrane fusion. Accordingly, the C-terminal domain of APOL1 can bind to the fusogenic protein VAMP8 [62], and in trypanosomes, a VAMP8 homologue (TbVAMP7B) is involved in APOL1 trypanosome-killing activity [63]. Furthermore, APOL3 can interact with the soluble-N-ethylmaleimide-sensitive-factor-attachment-receptor (SNARE) domain of VAMP8 or the R-SNARE helix of TbVAMP7B, and interaction of the APOL3 helices 4–5 and MAD1-2 with VAMP8 can promote the fusion between liposome vesicles respectively containing mitochondrial-like and endosomal-like membrane lipids [16]. In these experiments, APOL1 was unable to promote vesicle fusion with VAMP8-containing vesicles, unless a preincubation at low pH allowed its membrane insertion [16]. Thus, despite its membrane fusion potential, APOL1 cannot exert this activity unless transferred to acidic conditions. Accordingly, only APOL3, and not APOL1, was found to kill intracellular bacteria [13]. This process is likely to involve VAMP8 for bacterial digestion in endolysosomes.

STX17 was identified as VAMP8 partner for the fusion between autophagosomes and endolysosomes [64], but the VAMP8 partner for the fusion between mitophagosomes and endolysosomes remained unknown [65]. I propose that APOL3 fulfils this function (Fig. 4). APOL3 could be recruited to the mitophagosome membrane through interaction with cardiolipin [66, 67], which promotes APOL3/VAMP8-mediated fusion [16]. APOL3 shares several characteristics of STX17. Both proteins contain a double-stranded TM hairpin with high flexibility, due to an important proportion of helix-breaking amino acids [16]. Hydrophobic helix flexibility is crucial for the ability of TM peptides to drive membrane fusion [68], and may explain the detergent-like fusion activity of APOL3 on bacterial membranes [13]. Additional similarities between STX17 and APOL3 include Golgi-to-mitochondrion trafficking and activity in both mitochondrion fission and mitophagy initiation [59, 60, 69].

### APOL1 controls PI4KB and APOL3 traffic

APOL1 co-localizes with PI4KB and APOL3 in ATG9A vesicles [16], known to traffic PI4KB from the Golgi to the mitochondrion for autophagy initiation, which occurs together with mitochondrion fission at MERCSs [44, 70]. Moreover, APOL1 tightly associates with NM2A, the myosin known to be responsible for ATG9A vesicle trafficking [11, 71–73]. Finally, APOL1 can bind to prohibitin-2 (PHB2) [16], a mitophagy receptor involved in IFN-I-mediated autophagy together with GOLPH3 [74–76].

In APOL1 KO cells, Golgi PI4KB activity and actomyosin organization were not affected, indicating that APOL1 plays no significant role in PI4KB control at the Golgi [11]. However, in these cells mitochondrion fission and mitophagy were inhibited, suggesting that APOL1 is involved in both PI4KB and APOL3 transfer to the mitochondrion [16]. APOL1 association with NM2A may allow PI4KB/APOL3 traffic to the mitochondrion, and APOL1 binding to PHB2 could target PI4KB/APOL3 to MERCSs.

Consistent with these considerations, in APOL1 + 3 KO cells the mitochondrial phenotype linked to APOL3 deletion was also reverted [11], demonstrating the requirement of APOL1 for mitochondrial targeting of PI4KB. In these cells, the absence of APOL1 restored PI4KB activity at the Golgi, albeit at a slightly reduced level [11]. Therefore, preventing PI4KB delocalization still allows Golgi PI4KB activity despite APOL3 absence. Collectively, these observations suggest that the most important function of APOL3 is not PI4KB activity control, but PI4KB/NCS1 sequestration at the Golgi membrane, opposing to PI4KB/NCS1 delocalization by APOL1.

In APOL1Δ-expressing podocytes, PI4KB and NCS1 clearly delocalized from APOL3 and the Golgi, but remained strongly associated [11]. PI4KB and APOL1 are present in Golgi-derived ATG9A vesicles that are trafficking to various compartments such as MERCSs, the plasma membrane and endosomes [44, 77], and NCS1 was also detected in Golgi-derived vesicles trafficking to the plasma membrane [78]. Thus, through disruption of PI4KB-APOL3 interaction the C-terminal APOL1 variants may increase PI4KB and NCS1 trafficking to different membranes, including the plasma membrane, therefore inducing pleiotropic changes of membrane dynamics.

### The murine APOL3 and APOL1 homologues

**Fig. 5:**
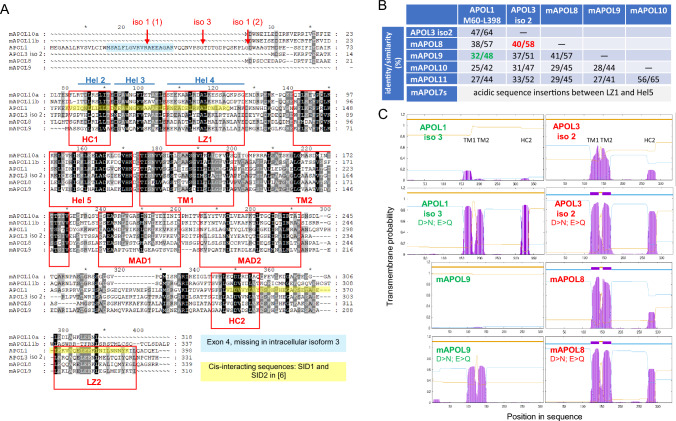
Murine APOL homologues of human APOL1 and APOL3. **A** Sequence alignment between human and mouse APOLs, using ClustalOmega (https://www.ebi.ac.uk/Tools/msa/clustalo/) for the alignment and GeneDoc (https://nrbsc.org/gfx/genedoc/) for the editing. The relative darkening reflects the level of similarity (black = high). Phylogenetic trees of these proteins are presented in [1, 2]. The N-terminal cleavage sites of different APOL1 isoforms (iso) are indicated by red arrows. The red boxes delimitate the various domains depicted in Fig. 1. Three helices defined in Ultsch et al. [17] are indicated in blue above the sequences. Stretches highlighted in light blue and yellow, respectively, represent a sequence missing in APOL1 isoform 3 and the APOL1 smallest interacting domains (SIDs) identified in Uzureau et al. [11]. **B** Percentages of sequence identity and similarity between human and mouse APOLs. The values obtained for the hypothetical APOL1 and APOL3 homologues in mice are coloured in green and red, respectively. In case of mAPOL7s, the extensive insertions of acidic stretches in the N-terminal domain clearly set these APOLs apart from APOL1 and APOL3. **C** Transmembrane probability in human and mouse intracellular APOLs, as defined by the TMHMM program (https://services.healthtech.dtu.dk/services/TMHMM-2.0/). Membrane insertion of the TM1 and TM2 helices has been experimentally demonstrated for both recombinant APOL1 and APOL3, although requiring acidic pH in case of APOL1 [12, 18]. To simulate acidic conditions, all D and E residues were converted into N and Q, respectively

Based on both overall sequence identity and relative pH dependence of transmembrane insertion, mAPOL8 and mAPOL9 are the best homologue candidates for APOL3 and APOL1 respectively (Fig. 5). Like APOL3, mAPOL8 could interact with NCS1, because mAPOL8 upregulation promotes synapse formation by neural stem cells [79], suggesting increased NCS1 activity [80]. Since the transgenic expression of the APOL1 G1 and G2 variants in mice can induce kidney disease [10], I propose that mAPOL8 is the mouse APOL family member inactivated by the APOL1 variants. Like APOL1, mAPOL9 is induced by IFN-I and is involved in restriction of viral infection [2, 81]. Moreover, like APOL1, mAPOL9 binds to PHB2 [2, 16]. However, contrasting with APOL1, mAPOL9 lacks a signal peptide, and is therefore only intracellular, in keeping with the primate specificity of APOL1 trypanosome-killing activity.

## Concluding remarks: kidney disease and other perspectives

The proposal that APOL3 and APOL1 control intracellular membrane dynamics provides explanations for kidney disease induced by APOL1 G1/G2 expression. Indeed, APOL3 inactivation by APOL1 C-terminal variants leads to drastic actomyosin reorganization, which influences kidney function notably through changes in podocyte shape and motility. I propose that APOL1 C-terminal variants can affect membrane dynamics in various cellular compartments, due to increased trafficking of PI4KB and NCS1 from the Golgi to other membranes. Changes in surface membrane dynamics could trigger effacement of podocyte foot processes and consecutive impairment of kidney activity. Kidney function is also particularly affected by interference with mitochondrial membrane fission [82], which reduces IFN-I-induced mitophagy and leads to mitochondrion dysfunctions together with exacerbated inflammatory reaction. Both effects on actomyosin organization and auto/mitophagy flux are characteristic of APOL1 variants-linked kidney disease, either genuine in humans or transgenic in mice or *Drosophila melanogaster* lines [10, 11, 83].

As a therapeutic perspective against this disease, disruption of APOL1 G1/G2 hydrophobic interactions with APOL3 could be attempted using either synthetic peptides of the interacting helices, lipid droplets [84] or overexpressed VAMP8 [85]. Alternatively, given the relative absence of dysfunctional phenotype in APOL1 KO cells [11, 16, 86], APOL1 inactivation could treat the disease [32, 87, 88]. However, APOL1 being involved in induction of mitophagy, APOL1 inactivation could reduce the ability to resist viral infection.

In addition to kidney disease, APOL1 or APOL3 variants could participate in other pathologies like neurotransmission disorders or cancer metastasis, which both involve NCS1 activity and membrane dynamics [3].

**Fig. 6:**
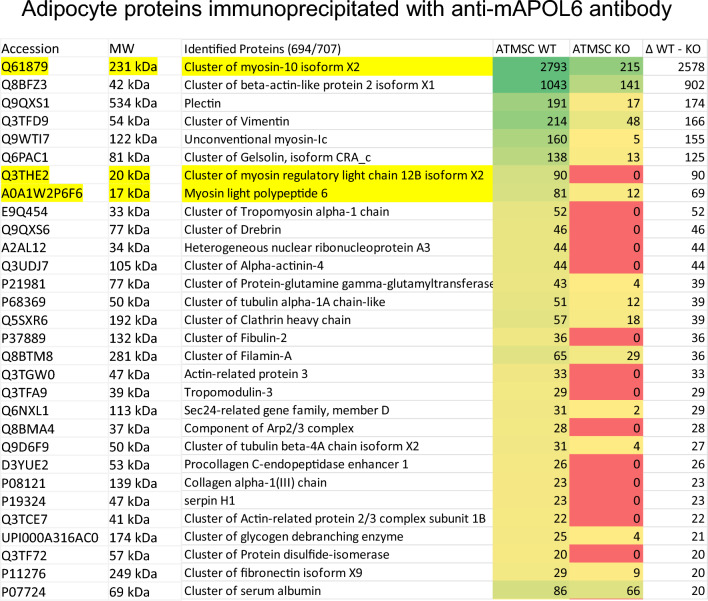
Association of mouse APOL6 with actomyosin components. List of the most abundant proteins identified by mass spectrometry of Adipose Tissue-Derived Mesenchymal Stem Cells (ATMSCs) immunoprecipitates from C57BL/6 WT or mAPOL6 KO mice, using rabbit antibodies raised against the mAPOL6 D36-K52 peptide. mAPOL6 KO mice were generated by flanking mAPOL6 exon 3, which contains the start codon, with inverted lox sites (PolyGene AG, Rümlang, Switzerland), followed by mice breeding with CMV-Cre mice and backcrossing into the C57BL/6 background for 10 generations. This resulted in exon 3 inversion and consecutive inhibition of mAPOL6 expression, verified by both (RT-)PCR and Western blotting (http://hdl.handle.net/2013/ULB-DIPOT:oai:dipot.ulb.ac.be:2013/279701.). The mAPOL6 KO mice exhibited reduced increase of both body weight and adipocyte lipid droplet size under high fat diet, particularly in males. The three myosin subunits are highlighted in yellow (Q61879 = heavy chain; Q3THE2 = regulatory light chain; A0A1W2P6F6 = essential light chain)

Possible future research directions also include the following:

- **Function of other APOLs?** Different APOL family members may play membrane-related functions in distinct organelles. For instance, in mouse lipid droplets, mAPOL6, a distant family member devoid of NCS1- and PI4KB-interacting helices, promotes triglyceride accumulation through the activity of its specific C-terminal region [89]. While involved in lipid droplet expansion under high fat diet, mAPOL6 strongly associates with myosin-10 (MYH10) and actomyosin components (Fig. 6), suggesting a function linking organelle size control with membrane dynamics (C. Vermeiren and E. Pays: http://hdl.handle.net/2013/ULB-DIPOT:oai:dipot.ulb.ac.be:2013/279701.). Consistently, MYH10 was found to govern both adipocyte adipogenesis and PI4P-mediated lipid droplet dynamics [90, 91]. In mAPOL7s, extensive acidic sequence insertions suggest a role in acidic environments, particularly under inflammatory conditions [20].
- **APOLs function in other membranes?** Apart from Golgi, mitochondrion and lipid droplet membranes, APOLs may also control the dynamics of the plasma membrane. This is suggested by the pleiotropic functions of APOL3-controlled NCS1, which affects surface channels and GPCR activities, exocytosis and synapse formation [33–40]. Moreover, since APOL3 is involved in mitophagosome fusion with endolysosomes, APOL3 could be involved in lysosome membrane dynamics, which is also supported by some PI4KB association with lysosomes [92]. Since virus-induced inflammation involves PI4KB-mediated transport of Stimulator of IFN Genes (STING) from Golgi to endosomes [93], APOLs could accompany PI4KB trafficking to endosomal membranes.
- **APOL1 interaction with NM2A?** The tight co-immunolocalization and stoichiometric immunoprecipitation of APOL1 with all three NM2A subunits myosin heavy chain 9 (MYH9), regulatory light chain (RLC) and essential light chain (ELC) (both light chains being NCS1-like proteins) suggest that APOL1 directly interacts with NM2A [11]. Accordingly, APOL1 can bind in vitro to the MYL12A RLC (J.H. Graversen and E. Pays, unpublished data).
- **APOL translocation across endosomal membranes?** Given the APOL similarities with SNARE proteins such as STX17, particularly in the flexible TM hairpin, it is relevant to note that membrane translocation has been reported for STX2 (epimorphin) and STX4 [94, 95]. Therefore, after endocytosis in trypanosomes APOL1 could hypothetically translocate from the luminal to the cytoplasmic face of the endosomal membrane to promote fusion between endosomes and mitochondrion, and/or between mitochondrial membranes (mitochondrion fenestration).
- **APOL control of gene expression?** PI4KB, APOL1 and APOL3 contain nuclear localization signals and are present in both nucleus and cytoplasm [11, 16, 96, 97]. Moreover, nuclear PI4KB and PI4P are associated with mRNA speckles and factors involved in pre-mRNA splicing or transport [98, 99], and both APOL1 and mAPOL9 immunoprecipitates contain RNA processing components [2, 11]. Thus, APOL1, APOL3 and PI4KB may influence gene expression. Consistently, NCS1 deficiency was reported to affect the mRNA levels of genes involved in mitochondrial activity [100].
- **APOL3 analogue in yeast?** Whereas NCS1 exhibits extensive structural and functional similarities with yeast FRQ1 [101], there is no yeast APOL homologue, suggesting that either FRQ1 activates PIK1 directly or FRQ1 binds to an APOL3-like protein. Along this line, *Saccharomyces cerevisiae* USE1 shares key APOL3 features: this protein with a highly flexible transmembrane span can interact with FRQ1, exhibits an unusual SNARE structure, and is involved in Golgi to endoplasmic reticulum vesicle traffic [102; https://yeastrc.org/pdr/viewY2HScreen.do?ID=97]. Interestingly, human USE1 can interact with APOL2 (https://www.ebi.ac.uk/intact/details/interaction/EBI-23699537).

## Data Availability

Not applicable.
